# Reliability and criterion validity of two applications of the iPhone™ to measure cervical range of motion in healthy participants

**DOI:** 10.1186/1743-0003-10-69

**Published:** 2013-07-05

**Authors:** Yannick Tousignant-Laflamme, Nicolas Boutin, Alexandre M Dion, Carol-Anne Vallée

**Affiliations:** 1 Université de Sherbrooke, School of Rehabilitation, 12e Avenue Nord, Sherbrooke, Qc 3001, Canada; 2 Centre de recherche Clinique Etienne-LeBel du CHUS, Sherbrooke, Qc, Canada

**Keywords:** Range of motion, Outcome measures, Reliability, Criterion validity, iPhone, Cervical spine, Digital inclinometer

## Abstract

**Summary of background data:**

Recent smartphones, such as the iPhone, are often equipped with an accelerometer and magnetometer, which, through software applications, can perform various inclinometric functions. Although these applications are intended for recreational use, they have the potential to measure and quantify range of motion. The purpose of this study was to estimate the intra and inter-rater reliability as well as the criterion validity of the *clinometer* and *compass* applications of the iPhone in the assessment cervical range of motion in healthy participants.

**Methods:**

The sample consisted of 28 healthy participants. Two examiners measured cervical range of motion of each participant twice using the iPhone (for the estimation of intra and inter-reliability) and once with the CROM (for the estimation of criterion validity). Estimates of reliability and validity were then established using the intraclass correlation coefficient (ICC).

**Results:**

We observed a moderate intra-rater reliability for each movement (ICC = 0.65-0.85) but a poor inter-rater reliability (ICC < 0.60). For the criterion validity, the ICCs are moderate (>0.50) to good (>0.65) for movements of flexion, extension, lateral flexions and right rotation, but poor (<0.50) for the movement left rotation.

**Conclusion:**

We found good intra-rater reliability and lower inter-rater reliability. When compared to the gold standard, these applications showed moderate to good validity. However, before using the iPhone as an outcome measure in clinical settings, studies should be done on patients presenting with cervical problems.

## Background

Cervical disorders are major health problems in our society and an important source of disability [1]. The mean prevalence of neck pain in the general population is 23.1% with a higher incidence noted in office and computer workers [2]. It is also one of the most common reasons to visit a health care professional [2]. Consequences of cervical disorders are multiple and include deficits such as pain and decreased range of motion (ROM) [3], which may reduce social participation and even lead to a sick leave [4].

Assessment of ROM is a significant part of the physical therapist’s role when evaluating a patient presenting with cervical disorders. Indeed, it helps to establish the clinical diagnosis and the prognosis, and also helps to elaborate an individualized treatment plan [5]. ROM is also an objective measure, which is essential to monitor the patient’s evolution throughout therapy. For these reasons, valid and reliable assessment tools are necessary.

Numerous valid tools are currently available to measure cervical ROM: they include inclinometers [6-8], digital inclinometers [9-13], measuring tape and goniometer [5], and the Cervical Range of Motion Device (CROM) [4,5,8,10,14-20]. The CROM is one of the most used tools among clinicians [21]. The systematic review of Williams et al. showed that the CROM has a good reliability for all cervical spine movements (ICCs = 0,58-0,99) and validity when compared to a gold standard (X-ray) (ICCs = 0,82-0,98). However, it is a relatively expensive instrument and only useful for the assessment of the cervical spine.

Regarding the digital inclinometers, only a few validity and reliability studies have been realized for cervical spine ROM [6-8]. Among digital inclinometers, the EDI-320 is an instrument that demonstrated good psychometric qualities (reliability ICCs = 0,69-0,96) according to two studies [4,18]. Unfortunately, this instrument is no longer available on the market. More recently, Prushansky *et al.*[22] showed that the conventional digital inclinometer has a good to excellent reliability (ICCs = 0,82-0,94). It also has good validity (ICCs = 0,62-0,83) when compared to the ultrasonography-based Zebris CMS 70P (Zebris Medizintechnik Gmbh, Isny, Germany). These authors measured cervical spine movements in three planes of movement and showed good results (ICCs = 0,82-0,94) with rotations in supine.

Recent smartphones are often equipped with an accelerometer (gravity sensor) and magnetometer (digital compass), which, through software applications, can perform various inclinometric functions. These applications are intended for recreational use, but have the potential to measure and quantify range of motion in many articulations, such as the cervical spine. For instance, previous studies have demonstrated the potential use of some applications in rehabilitation [23,24] and in ROM measurement [25]. The iPhone is easy to use and requires minimum training. Moreover, this instrument could allow the examiner (therapist) to obtain valid cervical ROM measurements, which can detect deficits in cervical ROM. Considering potential use of smartphones in rehabilitation and the favourable results obtained with digital inclinometers [13], the current study proposes to examine the psychometric properties of two applications (*clinometer* and *compass*) of the iPhone. The specific objectives are to determine the intra and inter-rater reliability of these two applications in the assessment of cervical ROM, as well as the criterion validity using CROM as the gold standard.

## Methods

### Design of the study

In this study, we used a descriptive correlational design to determine the reliability of the iPhone using intra and inter-rater reliability. For exploring the validity of these applications, we used criterion validity using the CROM as the gold standard. Because of the absence of any study on the reliability or validity of the iPhone for the measurement of cervical ROM, the population used in this study is composed of healthy participants (without neck pain and/or ROM deficits).

### Participants

Our sample consisted of 28 healthy volunteers (9 men and 19 women) aged from 19 to 43 years old (mean ± SD: 23 ± 6). Participants were included if they were 18 years of age or older and had neither cervical spine problem or neck pain. We excluded persons with cervical pathology (ex. painful diagnosis of arthritis or whiplash during the past year), psychiatric condition (ex. dementia, amnesia, delirium) or neurological disease (ex. Multiple sclerosis, Lou Gehrig’s Disease). The population included in this protocol was a convenient sample, recruited by purposive and snowball sampling. All volunteers consented for their participation in the study and did not receive monetary rewards or compensation for their time and participation to this study. The study was conducted in accordance with the Helsinki Declaration after approval from the ethics review board of the Centre hospitalier universitaire de Sherbrooke (project #10-199). All participants read the protocol, and a written consent was obtained in agreement with local ethics guidelines’. The study took place at the School of Rehabilitation of the Université de Sherbrooke. Considering the novel aspect related to the use of smartphones to measure ROM and the fact that we wanted to explore the psychometric properties of these applications, we opted for the recruitment of healthy subjects. All were assessed by the same instruments and the same observers.

### Instruments

#### iPhone’s applications

The iPhone is a smartphone with many possible applications. The application used to measure the cervical ROM in frontal and sagittal planes is *Clinometer (Peter Breitling, Version 3.3,*http://www.plaincode.com/products*)*, an application designed using the three inbuilt accelerometers (LIS302DL accelerometer). This application uses the internal three axes linear accelerometer to measure the direction of gravity’s pull. For this, the gyroscope stays in one position, no matter the orientation. When placed against a solid surface, the inclinometer compares the angle of the object to the gyroscope, and displays the results using the software interface.

**Figure 1:**
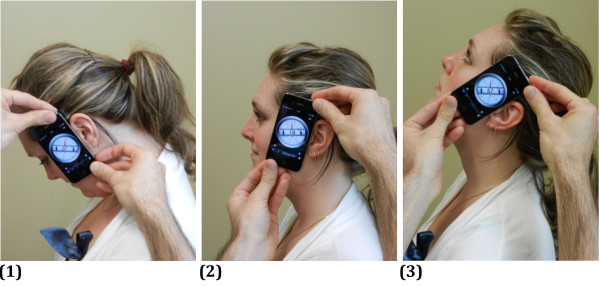
**Position of the iPhone for the measurement of flexion and extension. (1)** Position at end range of flexion. **(2)** Starting position. **(3)** Position at end range of extension. The side of the iPhone is aligned with the ear insertion to the head.

**Figure 2:**
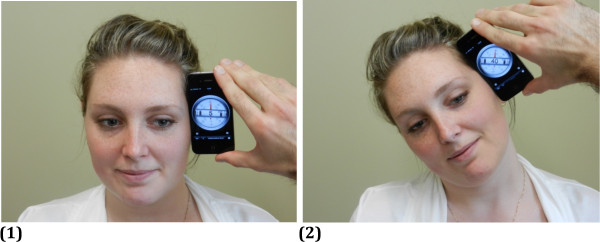
**Position of the iPhone for the measurement of right lateral flexion. (1)** Starting position. **(2)** Position at end range of right lateral flexion. The iPhone’s level is aligned with the corner of the eye.

Flexion/extension measures were taken with the iPhone placed on the left side of the head, aligned with the ear (see Figure 1). Left and right Side flexion were measured with the iPhone on contralateral head side with level aligned with the eyes (see Figure 2).

**Figure 3:**
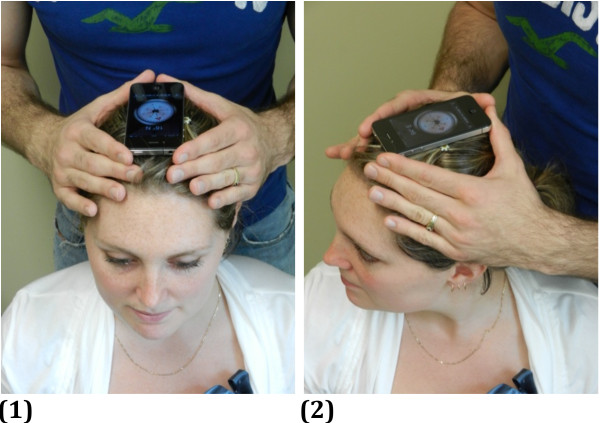
**Position of the iPhone for the measurement of right rotation. (1)** Starting position. **(2)** Position at end range of right rotation. iPhone’s cover is fixed on the head, compass aligned in front with the nose.

The application used to measure the cervical ROM in horizontal plane is *Compass,* software already integrated in the iPhone. In order to point out the orientation of the iPhone, the application uses the built-in magnetometer, which the senses orientation relative to the Earth’s magnetic field using the Hall effect (http://www.memsjournal.com/2011/02/motion-sensing-in-the-iphone-4-electronic-compass.html). The chip (AKM AK8975) senses the field in three directions, and from that can figure where the magnetic field pointing north is. Moreover, it also uses the accelerometer that tracks the movement of the device to measure changes in orientation. We choose the magnetic north to obtain our results. Rotation measures were taken with the iPhone placed on participant’s head with the arrow aligned with the nose (see Figure 3).

#### Cervical Range of Motion Device (CROM)

The CROM was used for the measurement of cervical flexion, extension, lateral flexions and rotations. This eyeglasses-like instrument has three inclinometers placed at three different positions: one near the left ear for flexion/extension (sagittal plan) and another for the lateral flexions on forehead (frontal plane) and both are gravity dependent. Finally, the one on the top of the head (horizontal plane) is used for the measurement of rotations; it is magnetic dependant, therefore, a magnetic brace must be placed around the neck. This instrument was used as our gold standard considering that its reliability and validity have been studied extensively [10,18,26,27].

### Procedures

#### Clinical procedures

For the purpose of this study, participants were simply asked to perform maximal (end-range) neck flexion, extension, left side flexion, right side flexion, left head rotation and right head rotation. Each participant was asked to perform neck movement at his/her own pace without going to fast.

#### Selection of examiners for the reliability study

Four students in physical therapy received three hours of training to adequately manipulate the CROM device. In their training, they also taught other classmates how to use the device during a two-hour session to enhance their competence in using the CROM. Following their training session, they determined which anatomical point of reference should be used with the iPhone and they trained for an hour to make sure their method was standardized. They then measured their own cervical ROM with the CROM and the iPhone (each student was measured twice). The intra-rater reliability was calculated for each student with the intraclass correlation coefficient (ICC). The two students with higher ICC’s results (ICC = 0,79 and 0,81) were assigned as examiner for the reliability part of the study. These two practiced their techniques in another two-hour session with four volunteers to standardize the procedure. Overall, the examiners had eight hours of training with the two instruments. This was done in order to minimize the error originating from examiners.

#### Selection of the examiner for the validity study

Between the two examiners, the one with the highest intra-rater reliability (highest ICCs) was chosen to undertake the validity study. This was done in order to minimize the error originating from examiners.

### Data collection

During all data collection sessions, the participants were instructed on the procedures. They were then asked to warm-up with five repetitions of all cervical movements. Afterwards, stabilizing straps were installed to prevent any trunk and shoulder movements during the movement’s execution (the same procedure were used during the selection of the examiners).

All measures were taken in the same order: flexion, extension, right and left lateral flexions and right then left rotations. This was done in order to minimize the possible bias induced by thixotropy [28].

For the sagittal and frontal plans, measures always corresponded to the total range (in degrees): the difference between final and initial measure. For example, a starting position of 5° slightly in extension and an end-range of 65° in flexion give us a total flexion of 70° (65° - (−5°) = 70°). Although the procedures for the CROM indicate to only take the final measure (angle at end-range), we could not use this method for the iPhone since our landmark was not necessary at 0° (i.e.: iPhone aligned with the ear), whereas it is always at 0° for the CROM. The total range (in degrees) was also used for the rotations movements.

#### Procedures for the reliability study

Two examiners entered two different rooms with a paired observer. They took all cervical ROM measures (flexion, extension, right then left lateral flexions and right then left rotations) with the iPhone while their paired observer wrote down the measures.

**Figure 4:**
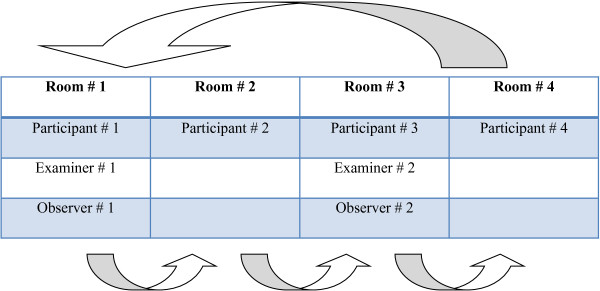
Data collection procedures.

The examiners then changed room in a clockwise motion until they had taken two measures of each movement for each participant with the iPhone. This allowed us to measure both intra and inter-rater reliability (see Figure 4).

#### Procedures for the validity study

Examiner 1 (which had the highest intra-rater ICCs during the selection of examiners process) entered room 1 with his paired observer. He took cervical ROM measures (flexion, extension, right then left lateral flexions and right then left rotations) with the CROM. The examiner then changed room in clockwise motion until he had taken a measure of each movement for each participant with the CROM; this allowed us to establish the criterion validity of the iPhone in comparison to this gold standard.

### Data analysis

#### Sample size calculation

In order to have minimal significant ICC value of 0.60 (1**-**β = 0.80; α = 0.05), a minimum of 20 subjects was required.

For the reliability part of the study, intra and inter-rater reliability were estimated with the intraclass correlation coefficient (ICC). ICC is a statistic designed to measure the size and direction of the association between two variables [29]. The values vary between −1 (perfect negative association) and +1 (perfect positive association). Different guidelines exist for the interpretation of ICC, but one reasonable scale is that an ICC value of less than 0,4 indicates poor reproducibility; ICC values in the range of 0,4 to 0,75 indicate fair to good reproducibility, and an ICC value of greater than 0,75 shows excellent reproducibility [30].

To estimate the criterion validity, we used the ICC and Pearson’s correlation coefficient. Some consider the ICC to be more accurate than Pearson’s correlation coefficient. For example, if one examiner always measures 5° more than another examiner, Pearson’s correlation coefficient would still be high. The ICC has the advantage to control for this bias, and for the later example, the ICC would be lower since it verifies if the values are the same and not only associated. The interpretation of the ICCs for the validity part of the study, an ICC value of less than 0,5 indicates poor validity; ICC values in the range of 0,5 to 0,65 indicate moderate to good validity, and an ICC value of greater than 0,65 shows good validity. Reference values [30] are reported in Table 1. Thereafter, 95% confidence intervals (95% CI) were constructed around the point estimated to account for sampling variation. Finally, descriptive statistics for measures of ROM (degrees) for each movement are reported for the iPhone and the CROM using mean and standard deviation.

**Table 1:** Reference values of the interpretation of ICCs

		**Good**	**Moderate**	**Poor**
**ICC**	**Reliability**	≥0.75	0.40-0.75	≤0.4
	**Validity**	>0.65	0.50-0.65	<0.50

## Results

### Intra-rater reliability

The highest ICCs were observed for examiner 1; they varied between 0.66-0.84; lower ICCs were found for examiner 2 where they varied between 0.17-0.68. Except for rotations, all movements had a good to excellent reliability, where side flexions demonstrated the best ICCs, and rotation the lowest ICCs. Table 2 shows the ROM obtained and the reliability coefficients (ICCs) for each movement.

**Table 2:** Intra-rater reliability and descriptive statistics for measurements of cervical spine ROM using the iPhone

	**Examiner 1 / iPhone 4**				**Examiner 2 / iPhone 3 GS**			
	**Trial 1 (Mean ± SD)**	**Trial 2 (Mean ± SD)**	**ICC**	**95% CI for ICC**	**Trial 1 (Mean ± SD)**	**Trial 2 (Mean ± SD)**	**ICC**	**95% CI for ICC**
Flexion	55,3° ± 9,9°	56,6° ± 8,2°	0,78	0,58-0,89	58,9° ± 8,1°	61,4° ± 8,0°	0,68	0,41-0,84
Extension	83,3° ± 15,2°	81,1° ± 14,6°	0,84	0,69-0,92	90,7° ± 15,9°	84,3° ± 13,2°	0,42	0,06-0,68
Right lateral flexion	44,6° ± 8,3°	46,5° ± 8,7°	0,77	0,56-0,89	52,0° ± 8,1°	41,5° ± 7,2°	0,68	0,42-0,83
Left lateral flexion	46,6° ± 7,1°	48,3° ± 7,6°	0,78	0,59-0,89	50,0° ± 6,2°	49,9° ± 6,7°	0,68	0,41-0,84
Right rotation	71,1° ± 15,8°	73,6° ± 13,4°	0,74	0,51-0,87	87,8° ± 31,0°	91,7° ± 27,6°	0,17	−0,21-0,5
Left rotation	74,5° ± 16,2°	76,2° ± 13,1°	0,66	0,39-0,83	87,6° ± 29,0°	76,3° ± 24,5°	0,28	−0,54-0,67

### Inter-rater reliability

To calculate the inter-rater reliability, we compared the average ROM value for each movement between both examiners. We found a moderate inter-rater reliability for movements in the sagittal (ICCs = 0.48-0.49) and frontal axis (ICCs = 0.40-0.54), but a poor inter-rater reliability in the transverse axis (ICCs = 0.07-0.09). The complete results are presented in Table 3.

**Table 3:** Descriptive and inter-rater reliability for measurements of cervical spine ROM using the iPhone

	**Examiner 1 (mean ± SD)**	**Examiner 2 (mean ± SD)**	**ICC**	**95% CI**
Flexion	56,0° ± 8,3°	60,1° ± 7,0°	0,48	0,14-0,72
Extension	82,2° ± 14,0°	87,5° ± 12,0°	0,49	0,15-0,72
Right lateral flexion	45,6° ± 7,8°	51,8° ± 7,0°	0,54	0,22-0,75
Left lateral flexion	47,5° ± 6,8°	50,0° ± 5,8°	0,40	0,04-0,67
Right rotation	72,4° ± 14,5°	89,7° ± 21,3°	0,09	−0,28-0,44
Left rotation	75,4° ± 14,7°	82,0° ± 22,3°	0,07	−0,30-0,42

### Criterion validity

When compared to our chosen gold standard (CROM), we observed good validity for the movements of flexion (ICC = 0,76; r = 0.69, p = 0.001), right lateral flexion (ICC = 0,85; r = 0.80, p < 0.001) and left lateral flexion (ICC = 0,70; r = 0.63, p < 0.001).

We observed moderate validity for the movement of extension (ICC = 0.58; r = 0.56, p = 0.002) and right rotation (ICC = 0.55; r = 0.58, p < 0.01). Finally, we found a poor validity for the movement of left rotation (ICC = 0.43; r = 0.38, p = 0.04). Table 4 shows the complete results of the criterion validity (ICCs as well as the Pearson’s correlation coefficient).

**Table 4:** Criterion validity of the iPhone compared to the CROM

	**iPhone (Mean ± SD)**	**CROM (Mean ± SD)**	**ICC ± [95% CI]**	**Pearson’s r (p-value)**
Flexion	56.0° ± 8.3°	57.1° ± 8.4°	0.76 [0.55-0.88]	0.69 p < 0.001
Extension	82.2° ± 14.0°	85.5° ± 12.3°	0.58 [0.27-0.78]	0.56 p = 0.002
Right lateral flexion	45.6° ± 7.8°	44.7° ± 8.2°	0.85 [0.70-0.93]	0.80 p < 0.001
Left lateral flexion	47.5° ± 6.8°	47.2° ± 7.0°	0.70 [0.46-0.85]	0.63 p < 0.001
Right rotation	72.4° ± 14.5°	73.8° ± 8.6°	0.55 [0.23-0.76]	0.58 P < 0.01
Left rotation	75.4° ± 14.7°	74.9° ± 8.6°	0.43 [0.08-0.69]	0.38 p = 0.04

## Discussion

This study is the first to examine the predictive value of two applications of the iPhone, which have the capability to measure cervical ROM using the CROM as the accepted gold standard. Although a few studies were already done on the validity of a digital device for the measurement of cervical ROM, no previous study was done on the digital inclinometer and/or the compass of the iPhone for the measurement of cervical ROM.

### Intra-rater reliability

Reliability estimates are very important psychometric properties since before an instrument can be considered valid, it needs to be reliable. The findings of this study showed that when the cervical ROM is measured with the iPhone by the same examiner (intra-rater reliability), similar results can be expected from one session to the next. It is possible to compare the current results of intra-rater reliability with digitals inclinometers such as Electronic Digital goniometer (EDI-320) for the measurement of active neck movement. For instance, in the sagittal plane, we found good reliability for flexion (ICC = 0,78; 95% CI: 0,58-0,89) and extension (ICC = 0,84; 95% CI: 0,69-0,92). Tousignant *et al.*[13] also reported good reliability for flexion (ICC = 0.77; 95% CI: 0,62-0,87) and extension (ICC = 0,83; 95% CI: 0,63-0,92) using the EDI-320. However, this device is not on the market anymore. In comparison, the reliability coefficients found in our study were slightly higher.

For the frontal plane, we found good intra-rater reliability (ICCs = 0.77-0.78; 95% CI: 0,56-0,89), while Prushansky *et al.*[22] found similar results with a digital inclinometer (ICCs = 0.82-0.90; 95% CI: 0,61-0,95).

Finally, in the transverse axis, we found moderate to good intra-rater reliability (ICCs = 0.66-0.74; 95% CI: 0,39-0,87), while Prushansky *et al.*[22] observed higher ICCs (ICCs = 0.84-0.92; 95% CI: 0,68-0,96). This might be explained by the fact that they took their measurements with the inclinometer while the subjects were in supine position, whereas we used the compass rather than the inclinometer of the iPhone. Since the compass is not influenced by gravity, but rather by orientation of the iPhones, it has more potential source of error than the inclinometer, which could have easily influenced the intra and inter-rater ICCs. Furthermore, the magnetometer which serves as the hardware for the compass application is more sensible of the presence of electro-magnetic fields which is another factor that could have contribute to the lower ICCs for the measurements of neck rotation.

### Inter-rater reliability

When the ROM measured by two independent examiners were compared, our ICCs were moderate for movements in the sagittal plane (ICCs = 0.48-0,49; 95% CI: 0,14-0,72) and in the frontal plan (ICCs = 0.40-0,54; 95% CI: 0,04-0,75). When we look closely at our results, we found that examiner 2, who used an iPhone generation 3GS, always had higher ROM measures than examiner 1, who used an iPhone generation 4. Considering that Apple uses an LIS302DL accelerometer for both iPhones 4 and 3GS and the two different generations of iPhone had the same operating system (iOS 4), factors related to the positioning of the iPhone might explain this observation. We also found poor correlation in transverse plan (ICC = 0.07-0,09; 95% CI: -0,30-0,44), which again might be explained by the presence of electro-magnetic fields that could influence the measure. On the other hand, it could also be attributed to the examiner since examiner 2 showed lower intra-rater reliability.

### Validity

Cervical ROM measured with the iPhone presented comparable results (moderate to good validity) when compared to the ROM measured with the CROM for all cervical movements, except for the movement of left rotation (ICC = 0.43). On the basis of this relation, the validity of the iPhone can be considered good for these movements for a same examiner, except for rotation. The poor results observed for the movements of rotation (ICC < 0.60) may partly be explained by the fact that it was measured by an application very sensible to electro-magnetic fields. This can lessen the accuracy of the, measurement. It could also be explained by the movement and/or positioning of the iPhone during the measurement of cervical rotation.

To our knowledge, no study examining the validity of the iPhone for assessing cervical ROM has been published. However, a recent article on the reliability and validity of a relatively inexpensive digital inclinometer reported results that were similar to our findings in sagittal and frontal planes: a good reliability (ICCs = 0,82-0,94) but lower validity (r = 0,62-0,83). Results were different for the rotation movements: a good reliability (ICCs = 0,84-0,92) and poor validity (results not reported). Their better results obtained for the reliability of rotations might be explain by the fact that rotations measured in supine position [22].

Our results show that measures of extension and right rotation had poor inter-rater reliability and thus mined the validity of this measure. This discrepancy may be attributed to the data collection procedures or the placement of the iPhone on the top of participant’s head. Special efforts were made in this study to minimize this type of error, but we suggest that future measurements of rotation movements might be done with the iPhone on the top of the forehead while the person is lying supine as done by Prushansky [22].

### Strengths and limitations

First, the two examiner’s initial preparation (training) with the CROM represents strength. The assessment of the examiner’s skills showed that they were competent (ICC > 0,65) in the use of the method and the device (E1: ICC = 0,81; 95% 0,56-0,92. E2: ICC = 0,79; 95% 0,52-91) (see Table 1 for ICC reference values). For the validity study, we purposely chose examiner 1 in order to minimize the source of error coming from the examiner.

Second, standardization of the procedures also helped minimize random errors. To achieve this, all participants were stabilized in order to avoid compensation. Also, the research assistant always gave the same instructions before each measurement for all participants and the environment was identical during all the data collection process: same rooms, same orientation or the participants (facing east), same chairs, etc.

Thirdly, measures were taken with the iPhone and the CROM were always taken in the same order. Thus, if the cervical ROM increased with repetitions, the pattern would be the same for all participants and would not influence our results.

Finally, the iPhone measures were always taken before the CROM measure to prevent an information bias. Due to the numerous measurements took with the iPhone, we considered that it would have been impossible for the examiner to remember all the results and influence its readings using the CROM. Therefore, we think that this help minimized an information bias.

This study also had limitations. First, data was collected on a sample of healthy participants, which limits the external validity. Although we tried to minimize bias affecting the internal validity, but the fact that examiner 1 had higher intra-rater ICCs than examiner 2 might partly explain the modest results for the inter-rater reliability.

## Conclusion

Implications of this study relate to the use of the iPhone to measure the cervical ROM in patients without neck dysfunction. The iPhone is a popular device and has good potential for clinical use. This instrument is easy to use and requires minimum training. Moreover, this instrument could allow the examiner (therapist) to obtain valid cervical ROM measurements, which can detect deficits in cervical ROM.

In the current non-probabilistic sample of healthy participants, we found that the iPhone had good intra-rater reliability but lower inter-rater reliability. When compared to a gold standard (CROM), the iPhone showed moderate to good validity for movements in the sagittal and frontal plans, but poor validity for rotation movements. At this stage, we cannot recommend the use of the iPhone to measure cervical range of motion in all directions. Moreover, before using the iPhone as an outcome measure in clinical settings, we should focus on finding better positioning method for the measurement of cervical rotation and more importantly, studies should be done on patients presenting with cervical problems.

## Competing interests

The authors declare that they have no competing interest.

## Authors’ contribution

NB, AD and CAV participated in the study’s design and coordination, took all measures, participated in the statistical analysis and draft the manuscript. YTL supervised all steps of the project, was responsible for the design of the study, had major contributions in the preparation of the manuscript and gave methodological advices during the entire research process. All authors read and approved the final manuscript.

## Authors’ information

At the time of data collection, NB, AD and CAV are students were final year physical therapy students at the Université de Sherbrooke. YTL is an associate professor at the School of rehabilitation of the Université de Sherbrooke and is a supported member of the Centre de recherche Clinique Etienne-LeBel du CHUS.

## References

[B1] Dvir Z Prushansky T Reproducibility and instrument validity of a new ultrasonography-based system for measuring cervical spine kinematics Clin Biomech 2000 15 9 658 664 10.1016/S0268-0033(00)00033-4 10946098

[B2] Hoy DG Protani M De R Buchbinder R The epidemiology of neck pain Best Pract Res Clin Rheumatol 2010 24 6 783 792 10.1016/j.berh.2011.01.019 21665126

[B3] Buskila D Sarzi-Puttini P Ablin JN The genetics of fibromyalgia syndrome Pharmacogenomics 2007 8 1 67 74 10.2217/14622416.8.1.67 17187510

[B4] Lee H Nicholson LL Adams RD Cervical range of motion associations with subclinical neck pain Spine 2004 29 1 33 40 10.1097/01.BRS.0000103944.10408.BA 14699273

[B5] Reese NB Bandy WD Joint Range of motion and muscle lenght testing 2002 St-Louis, Missouri: Saunders Elservier

[B6] Bush KW Collins N Portman L Tillett N Validity and intertester reliability of cervical range of motion using inclinometer measurements The Journal of Manual & Manipulative Therapy 2000 8 2 52 61 10.1179/10669810079081954623820304

[B7] Tucci SM Hicks JE Gross EG Campbell W Danoff J Cervical motion assessment: a new, simple and accurate method Arch Phys Med Rehabil 1986 67 4 225 230 3964055

[B8] Hole DE Cook JM Bolton JE Reliability and concurrent validity of two instruments for measuring cervical range of motion: effects of age and gender Man Ther 1995 1 1 36 42 10.1054/math.1995.0248 11327793

[B9] Mayer T Brady S Bovasso E Pope P Gatchel RJ Noninvasive measurement of cervical tri-planar motion in normal subjects Spine 1993 18 15 2191 2195 10.1097/00007632-199311000-00007 8278830

[B10] Love S Gringmuth RH Kazemi M Cornacchia P Schmolke M Interexaminer and intraexaminer reliability of cervical passive range of motion using the CROM and Cybex 320 EDI J Can Chiropr Assoc 1998 42 4 222 228

[B11] Hoving JL Pool JJ Mameren H Deville WJ Assendelft WJ Vet HC Winter AF Koes BW Bouter LM Reproducibility of cervical range of motion in patients with neck pain BMC Musculoskelet Disord 2005 6 59 10.1186/1471-2474-6-59 16351719 PMC1343553

[B12] Zwart JA Neck mobility in different headache disorders Headache 1997 37 1 6 11 10.1046/j.1526-4610.1997.3701006.x 9046716

[B13] Tousignant M Boucher N Bourbonnais J Gravelle T Quesnel M Brosseau L Intratester and intertester reliability of the cybex electronic digital inclinometer (EDI-320) for measurement of active neck flexion and extension in healthy subjects Man Ther 2001 6 4 235 241 10.1054/math.2001.0419 11673934

[B14] Nilsson N Christensen HW Hartvigsen J The interexaminer reliability of measuring passive cervical range of motion, revisited J Manip Physiol Ther 1996 19 5 302 305 8792318

[B15] Olson SL O’Connor DP Birmingham G Broman P Herrera L Tender point sensitivity, range of motion, and perceived disability in subjects with neck pain J Orthop Sports Phys Ther 2000 30 1 13 20 10705592 10.2519/jospt.2000.30.1.13

[B16] Peolsson A Hedlund R Ertzgaard S Oberg B Intra- and inter-tester reliability and range of motion of the neck Physiother Can 2000 52 3 233 242

[B17] Rheault W Albright B Beyers C Franta M Johnson A Skowronek M Dougherty J Intertester reliability of the cervical range of motion device J Orthop Sports Phys Ther 1992 15 3 147 150 18796788 10.2519/jospt.1992.15.3.147

[B18] Tousignant M Ld B O’Donoughue S Grahovac S Criterion validity of the cervical range of motion (CROM) goniometer for cervical flexion and extension Spine 2000 25 3 324 330 10.1097/00007632-200002010-00011 10703104

[B19] Youdas JW Carey JR Garrett TR Reliability of measurements of cervical spine range of motion--comparison of three methods Phys Ther 1991 71 2 98 104 1989013 10.1093/ptj/71.2.98

[B20] Youdas JW Garrett TR Suman VJ Bogard CL Hallman HO Carey JR Normal range of motion of the cervical spine: an initial goniometric study Phys Ther 1992 72 11 770 780 1409874 10.1093/ptj/72.11.770

[B21] Williams MA McCarthy CJ Chorti A Cooke MW Gates S A systematic review of reliability and validity studies of methods for measuring active and passive cervical range of motion J Manip Physiol Ther 2010 33 2 138 155 10.1016/j.jmpt.2009.12.009 20170780

[B22] Prushansky T Deryi O Jabarreen B Reproducibility and validity of digital inclinometry for measuring cervical range of motion in normal subjects. Physiotherapy Research International The Journal For Researchers And Clinicians In Physical Therapy 2010 15 1 42 48 10.1002/pri.443 19554615

[B23] Lee BC Kim J Chen S Sienko KH Cell phone based balance trainer J Neuroeng Rehabil 2012 9 10-0003-9-10 10.1186/1743-0003-9-10 22316167 PMC3340298

[B24] Wu HH Lemaire ED Baddour N Change-of-state determination to recognize mobility activities using a BlackBerry smartphone Conference proceedings: Annual International Conference of the IEEE Engineering in Medicine and Biology Society. IEEE Engineering in Medicine and Biology Society.Conference 2011 5252 5255 10.1109/IEMBS.2011.6091299 22255522

[B25] Shin SH du Ro H Lee OS Oh JH Kim SH Within-day reliability of shoulder range of motion measurement with a smartphone Man Ther 2012 17 4 298 304 10.1016/j.math.2012.02.010 22421186

[B26] Audette I Dumas J JC N SDS J Validity and between-day reliability of the cervical range of motion (CROM) device J Orthop Sports Phys Ther 2010 40 5 318 323 20436238 10.2519/jospt.2010.3180

[B27] Tousignant M Smeesters C Breton A Breton Ã Corriveau H Criterion validity study of the cervical range of motion (CROM) device for rotational range of motion on healthy adults J Orthop Sports Phys Ther 2006 36 4 242 248 16676874 10.2519/jospt.2006.36.4.242

[B28] Lieber RL Bodine-Fowler SC Skeletal muscle mechanics: implications for rehabilitation Phys Ther 1993 73 12 844 856 8248293 10.1093/ptj/73.12.844

[B29] Polgar S Thomas SA Introduction to research in the health science 2008 fifth edition Philadelphia: Churchill-Livingstone Elsevier

[B30] Fleiss JL Design and analysis of clinical experiments 1999 New York: Willey Classical Library

